# Detection of *stx_1_* and *stx_2_* Genes in Pennsylvanian White-Tailed Deer

**DOI:** 10.3390/toxins3060640

**Published:** 2011-06-16

**Authors:** Whitney M. Kistler, Surafel Mulugeta, Steven A. Mauro

**Affiliations:** 1 Southwestern Cooperative Wildlife Disease Study, University of Georgia, Athens, GA 30602, USA; Email: wkistler@uga.edu; 2 Department of Biological Sciences, Mercyhurst College, Erie, PA 16546, USA; Email: smulug75@lakers.mercyhurst.edu

**Keywords:** Shiga toxin, white-tailed deer, quantitative PCR

## Abstract

Shiga toxin-producing *E. coli* carrying the *stx_1_* and/or *stx_2_* genes can cause multi-symptomatic illness in humans. A variety of terrestrial and aquatic environmental reservoirs of *stx* have been described. Culture based detection of microbes in deer species have found a low percentage of samples that have tested positive for Stx-producing microbes, suggesting that while deer may contain these microbes, their overall abundance in deer is low. In this study, quantitative PCR (qPCR) was utilized to test for the presence of *stx* genes in white-tailed deer fecal matter in western Pennsylvania. In this culture independent screening, nearly half of the samples tested positive for the *stx_2_* gene, with a bias towards samples that were concentrated with *stx_2_*. This study, while limited in scope, suggests that deer may be a greater reservoir for *stx* than was previously thought.

## 1. Introduction

Bacteria are capable of producing bacteriophage-encoded Shiga toxin (Stx) proteins, which can cause illness in humans [1]. Based on amino acid similarity, these proteins are separated into two main types, Shiga toxin 1 (Stx1) or Shiga toxin 2 (Stx2). While Stx2 tends to be more pathogenic in humans in comparison to Stx1, bacteria expressing one or both types can produce symptoms ranging from mild diarrhea to hemolytic uremic syndrome, contributing to 100,000 cases of illness in the United States annually, which can carry a mortality rate of 10% among young children and the elderly [2,3].

*Escherichia coli* (*E. coli*) O157:H7 is the most intensely studied bacterial source of Stx. This bacterial species is responsible for an estimated 63,000 illnesses in the United States annually [4]. Various terrestrial and aquatic environments that serve as reservoirs for *E. coli* O157 and other microbes which contain *stx* genes have been identified [2]. While most studies have focused on cattle as the major reservoir for Stx-producing *E. coli* (STEC), transmission of *E. coli* O157:H7 from deer to human has also been implicated in outbreaks of sickness in humans [5]. Hence, deer may also be an ecological reservoir for bacteria that produce Stx and cause human disease. 

In support of the idea of deer serving as a reservoir for Stx-producing organisms, STEC has been isolated from deer feces [6,7]. However, studies that have tested for the presence of *E. coli* O157:H7 serotypes in deer feces suggest that the prevalence of this microbe is low, ranging in frequency from 0.25%–2.5% of the samples tested [8,9,10]. Taken together, these studies would suggest that while deer contain Stx-producing organisms such as *E. coli* O157:H7, they are not a major terrestrial reservoir for these microbes. 

However, nearly 500 other *E. coli* serotypes [11] and several non-*E. coli* bacteria [12,13] are capable of producing Stx, making it likely that the detection of *E. coli* O157 alone will produce an underestimate of the presence of Stx-producing bacteria in a sample. Since so many different types of bacteria can produce Stx, a quantitative PCR (qPCR) approach was employed in this study to detect the presence and relative abundance of *stx* genes in white-tailed deer fecal samples obtained from western Pennsylvania. These results, while limited in scope, provide evidence that deer might be a greater reservoir for Stx-producing microbes than has been previously thought. 

## 2. Materials and Methods

### 2.1. Sample Collection

Fifty fecal samples of white-tailed deer, *Odocoileus virginianus*, were collected on the forest floor in western Pennsylvania using sterilized equipment and collection bottles. To minimize collecting samples from the same deer, sample location and day of collection were varied over a 12 month period from four different locations. The first site (42°06'56.39'' N 80°09'09.22'' W) is a highly frequented State Park, the second and third sites (42°50'11.65'' N 80°18'00.25'' W and 41°50'11.65'' N 79°20'49.98'' W) are small immature forested areas in rural settings, while the fourth site (40°16'42.29'' N 79°45'52.11'' W) is a mature forested area. All fecal samples were taken from the surface of the fecal deposit, which were free of debris and were sufficiently hydrated and not crusted over, which we interpreted as being fresh fecal samples that were deposited on or close to the day of collection. All samples were processed within a 24 h period following collection. 

### 2.2. Sample Processing

Following collection, 0.4 grams of each fecal sample was placed in 50 mL TBS (10 mM Tris, pH = 8.0 and 50 mM NaCl). Bacteria were released from the fecal solid into the TBS solution by vortexing until fecal matter was homogenized in solution. Following bacterial release, the remaining fecal solid was allowed to sediment for 15 min. and 15 mL of the TBS/bacterial solution was used for DNA isolation. 

### 2.3. DNA Isolation

To isolate bacteria, the TBS/bacterial mixture was filtered onto a 47 mm mixed cellulose ester filter with a pore size of 0.45 µm. The filter was then placed in an eppendorf tube containing 600 µL of TE (10 mM Tris pH 7.5, 0.5 mM EDTA) solution and DNA was isolated following RNase digestion at a final concentration of 50 µg/mL, phenol:chloroform extraction and ethanol precipitation as described previously [14]. Final precipitated pellets were resuspended in 50 µL of a 10 mM Tris (pH = 7.5) solution. To avoid cross contamination of samples, initial processing was performed under a laminar flow hood and sterile technique was used throughout the procedure. 

### 2.4. Quantitative PCR

Of the 50 µL of purified DNA for each sample, 2.5 µL was subject to amplification for 45 cycles (95 °C denaturing for 15 seconds and 60 °C for 60 seconds) in an Applied Biosystems 7300 Real-Time PCR System for the *stx_1_* gene: Forward primer = GACTGCAAAGACGTATGTAGATTCG, Reverse primer = ATCTATCCCTCTGACATCAACTGC, fluorescent probe = TGAATGTCATTCGCTCTGCAATAGGTACTC), the *stx_2_* gene (Forward primer = ATTAACCACACCCCACCG, Reverse primer = GTCATGGAAACCGTTGTCAC, fluorescent probe = CAGTTATTTTGCTGTGGATATACGAGGGCTTG), and the *eae* gene (Forward primer = GTAAGTTACACTATAAAAGCACCGTCG, Reverse primer = TCTGTGTGGATGGTAATAAATTTTTG, fluorescent probe = AAATGGACATAGCATCAGCATAATAGGCTTGCT), which were all designed in a previous study [15]. The reaction conditions included incubation with a PCR buffer at final weights or concentrations of Tris (12 mM, pH = 7.0), KCl (50 mM), MgCl_2 _(5 mM), trehalose (0.15 mg), glycerol (1.6%), Tween-20 (0.2%), ultrapure bovine serum albumin (200 µg/mL), deoxyribounucleotides (125 µM of each nucleotide), and AmpliTaq Gold DNA polymerase (0.5 U) . Each signal was quantified by measuring the release of a fluorophore (FAM for *stx_1_* and ROX for *stx_2_* and *eae*) from the five prime end of the fluorescent probe from the quencher (BHQ-1 for *stx_1_* and BHQ-2 for *stx_2_* and *eae*) at the three prime end of the same probe. To quantify each signal, the fluorescence intensity from each positive reaction was normalized to a standard curve of known concentrations of DNA from a positive control reference laboratory strain for *stx_1_* and ATCC bacterial stock 43889 for *stx_2_*, final values which were converted to gene copies of each stx variant per gram of fecal matter originally used for DNA isolation. A sample was considered to be positive for a particular gene when the CT value was two orders of magnitude greater than the CT value of a 10 mM Tris no template control. Additionally, all positive signals were confirmed by gel electrophoresis, which gave a single band of the predicted size for all positive samples. Negative inhibition of PCR signal was not noted in this study. This was assessed by doping a known concentration of a positive control (previously described) into a fraction of the isolated DNA from a sample, which was then followed by qPCR for the gene of interest. The resulting concentration of the positive control, determined by subtracting the concentration of the gene of interest that was already in the sample from the total concentration in this experiment, was not different from the concentration of the positive control at the same concentration that was not doped into a fraction of the sample.

## 3. Results and Discussion

Fifty fecal samples from white-tailed deer in western Pennsylvania were collected and analyzed for the presence of the *stx_1_* or *stx_2_* genes using qPCR with primers that are specific for these genes. These results are summarized in Table 1. While both *stx_1_* and *stx_2_* genes were detected, the presence of *stx_2_* was detected with a higher frequency in comparison to *stx_1_*, present in 23/50 (46%) of the samples tested in comparison to the 5/50 (10%) of the samples that tested positive for *stx_1_* (Table 1). All of the samples which tested positive for the *stx_1_* gene also tested positive for the *stx_2_* gene. The *eae* gene, which is used here as a marker for *E. coli* O157 or non-O157 *E. coli* that have an enhanced potential to cause human illness, was coincident with *stx_1_* or *stx_2_* in only 4/50 (8%) of the samples tested. 

**Table 1:** Fraction of deer fecal samples that contained *stx* genes.

Gene Tested	Fraction (%) Positive for Gene
*stx_1_*	5/50 (10.0)
*stx_2_*	23/50 (46.0)
*stx_1_ + stx_2_*	5/50 (10.0)
*stx_1_ or stx_2_ + eae*	4/50 (8.0)

**Figure 1:**
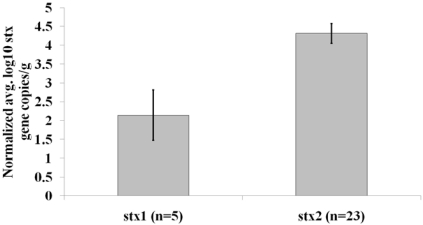
Average normalized logarithmic concentration of *stx* genes/g of fecal matter. The sample size for each average value is shown in parentheses and standard error bars are indicated. The *p*-value calculated from a Mann-Whitney U test in a comparison between sample types was 0.0023.

The concentration of either the *stx_1_* or *stx_2_* gene within a sample was variable. For example, the concentration range for *stx_1_* in a sample was as low as nine gene copies/g of feces sampled in one sample, which was approximately the lower limit of detection using qPCR in this study, to as high as 760 gene copies/g of fecal matter in another sample. For *stx_2_*, the range was even greater, between 77 to greater than two million gene copies/g of fecal matter between the least and highest concentrated sample analyzed. The average normalized *stx_2_* DNA concentration in fecal samples testing positive for the *stx_2_* gene was 4.3 log10 gene copies/g of feces, while the average normalized concentration of the stx1 gene was only 2.1 log10 gene copies/g of feces in samples positive for this *stx* variant, an over 200-fold difference in concentration between the two *stx* types analyzed (Figure 1).

In this study, nearly half of the samples collected tested positive for *stx* DNA, which is a considerably higher percentage of detection frequency compared to previous work that has focused on culture based assays to detect the presence of STEC [8,9,10]. In similar approaches analyzing cow feces, reports vary from anywhere between 18%–82% of fecal samples testing positive for a *stx* gene [16,17,18,19,20,21]. Our results certainly fall within this same range of *stx *detection frequency, which suggests that deer can act as a reservoir for *stx*-producing organisms to the same extent that cows do. 

The *stx_2_* gene was present in much higher concentration in comparison to *stx_1 _*in the deer fecal samples tested in this study. This indicates that microbes that produce Stx2 may be the predominant type of Stx-producing organism capable of being transmitted from deer to the environment. In support of this idea, studies that probed for *stx* genes in STEC isolates originating from deer showed a much higher frequency of *stx_2_* than *stx_1 _* [6,7]. Stx2 is more commonly associated with severe pathologic states in humans compared to those individuals infected with strains that produce Stx1 [1]. Taken together, these results seem to indicate that the pathogenic potential of *stx-*producing organisms resulting from deer could be high. However, it is noteworthy that the primers used in this study did not discriminate between the different *stx2* subtypes known to exist, which differ in their pathogenic potential [2]. Moreover, the coincidence of the *eae* gene, which is used here as a marker for the presence of *E. coli *with an enhanced capability to cause human illness, with *stx_2_* in these samples was low. Thus, while the overall presence of the *stx_2_* gene in deer feces can be high, the ability of microorganisms harboring this gene in deer feces to produce a functional Stx2 protein remains in question. Based on the low abundance of STEC isolated from deer feces in other studies, a potential explanation for the high percentage of samples that test positive for *stx_2_* in our study is that other bacterial species known to express *stx_2_* reside in deer fecal samples [8,9,10,12,13]. Studies aimed at isolating and characterizing such strains should be an exciting area of future research that will allow for a better understanding of the role deer have as a reservoir for Stx-producing microbes.

## 4. Conclusions

There are an estimated 15 million free ranging white-tailed deer in the United States [22]. In Pennsylvania alone, hunters harvested over 300,000 deer in the 2007/2008 hunting seasons [23], providing ample opportunity for transmission of Stx-producing bacteria originating from deer to humans to occur. Moreover, the presence of Stx-producing bacteria in deer fecal matter has the potential to contaminate nearby bodies of water and food sources due to runoff following rain events, thereby indirectly impacting human health. Our results implicate deer as a greater reservoir for Stx-producing organisms than has been previously thought. This, coupled with the potential of deer to spread these organisms in nature, demonstrates that there is a need to further investigate the role deer have in harboring and spreading Stx-producing organisms in the environment. 
